# Characterization of gut dominant microbiota in obese patients with nonalcoholic fatty liver disease

**DOI:** 10.3389/fcimb.2023.1113643

**Published:** 2023-01-23

**Authors:** Li-ting Jin, Ming-Zhi Xu

**Affiliations:** ^1^Zhejiang University of Medicine, Hangzhou, Zhejiang, China; ^2^Department of General Medicine, Zhejiang Cancer Hospital, Institute of Basic Medicine and Cancer (IBMC), Chinese Academy of Sciences, Hangzhou, Zhejiang, China; ^3^Department of Endocrinology and Metabolic Disease, Shulan (Hangzhou) Hospital Affiliated to Zhejiang Shuren University Shulan International Medical College, Hangzhou, Zhejiang, China

**Keywords:** *Faecalibacterium prausnitzii*, lymphocytes, microbiota interaction, pathogenic factor, disease prevention

## Abstract

In obese patients, non-alcoholic fatty liver (NAFLD) is common. However, whether there is a connection between the gut microbiota and the onset of NAFLD in obese people is yet unknown. Using quantitative real-time PCR, the microbiota of feces of the eligible 181 obese individuals was identified to compare the differences in gut microbiota between obesity with NAFLD and simple obesity. According to the findings, the gut dominant microbiota was similar between obesity with NAFLD and simple obesity. Nonetheless, compared to the simple obesity group, the quantity of *Faecalibacterium prausnitzii* colonies was much lower in the obesity with the NAFLD group. *Bacteroides* were present in greater than 65% of both groups. *Bacteroides*, *Clostridium leptum*, and *Clostridium butyricum* accounted for more than 80% of the cases in the obesity with NAFLD group, whereas *Bacteroides*, *Clostridium butyricum*, and *F. prausnitzii* accounted for more than 80% of the cases in the simple obesity group. We look for potential contributing variables to obesity-related NAFLD and potential prevention measures for obese people. Based on a multi-factor logistic regression analysis, lymphocytes may be a risk factor for obesity with NAFLD while *F. prausnitzii* may be a protective factor. Additionally, *F. prausnitzii* is positively impacted by *Bacteroides*, *Clostridium leptum*, *Clostridium butyricum*, and *Eubacterium rectale*, yet adversely impacted by Enterobacteriaceae. Notably, lymphocytes and *F. prausnitzii* may help determine whether obese patients would develop NAFLD.

## Introduction

A worldwide epidemic of obesity has emerged, and obesity raises the risk of diabetes, heart disease, and cancer (Liu et al., 2017; Rosengren, 2021), leading to a sharp rise in medical expenses. The normal human gastrointestinal tract has more than 1000 species of microbiota, many of which are crucial to human health (Gilbert et al., 2018; Fan and Pedersen, 2021; Hou et al., 2022). Recently, research suggests that disorders of gut microbiota may be a causal factor in a variety of metabolic diseases (Geach, 2017; Liu et al., 2017; Friedman et al., 2018; Sun et al., 2018; Haghikia et al., 2022; Olofsson and Bäckhed, 2022). The obese population differed from the normal population in gut microbiota, showing a decrease in Clostridia and an increase in Desulfovibrio (Wang and Hooper, 2019). While there is a discrepancy in the Bacteroidetes, some claim that Bacteroidetes are declining (Liu et al., 2017), but others claim Bacteroidetes are rising (Vieira-Silva et al., 2020). Obesity may be brought on by gut microbiota (Meijnikman et al., 2018). Weight loss can also result from altering the gut microbiota; for example, supplementing with *Pasteurized A. muciniphila* reduced body weight, fat mass, and hip circumference (Depommier et al., 2019).

The gut microbiota of people with NAFLD has unique traits. NAFLD exhibited a significant increase in *Escherichia*, *Dorea*, and *Peptoniphilus* increase (Del Chierico et al., 2017; Hoyles et al., 2018), a decrease in the *Bacteroides* (Cheng et al., 2022), *F. prausnitzii *(Pettinelli et al., 2022), *Eubacterium* and *Prevotella* (Del Chierico et al., 2017; Hoyles et al., 2018). Gut microbiota is associated with the severity of NAFLD (Dong and Jacobs, 2019), NAFLD progressing to nonalcoholic steatohepatitis (NASH) exhibited a significant increase in the abundance of the Proteobacteria phylum, a decrease in the Firmicutes phylum (Loomba et al., 2019). Gut microbiota can also cause NAFLD (Chiu et al., 2017; Aron-Wisnewsky et al., 2020a; Nagashimada and Honda, 2021; Bauer et al., 2022). By utilizing probiotics or synbiotics, patients with NAFLD may even experience a reduction in steatosis and fibrosis (Xie and Halegoua-DeMarzio, 2019; Aron-Wisnewsky et al., 2020b; Khan et al., 2021). Yet, the mechanisms by which gut microbiota cause NAFLD and exacerbate NAFLD are not yet clear.

NAFLD has attracted attention in recent years. About one-fourth of adults worldwide are affected by NAFLD (Bauer et al., 2022). The pathology of NAFLD is characterized by steatosis. NAFLD and obesity are strongly linked (Aron-Wisnewsky et al., 2020a). NAFLD can progress to NASH, and further fibrosis can lead to cirrhosis or even liver cancer, posing a threat to people’s health. Detailed studies on the gut dominant microbiota characteristics of obesity with NAFLD have not been performed. This study intends to explore the gut dominant microbiota characteristics, and the risk and protective factors for obesity with NAFLD, to provide ideas for the prevention of co-occurring NAFLD in obese patients.

## Materials and methods

### Ethics approval and inclusion criteria

This study was reviewed and approved by the ethics committee of Shulan (Hangzhou) Hospital Affiliated to Zhejiang Shuren University Shulan International Medical College (accession number KY2022103). All patients/participants signed informed consent.

We retrospectively collected clinical information on obese patients (BMI ≥ 28 kg/m^2^) performed gut dominant flora testing, and hospitalized in endocrinology, hepatology, cardiovascular, physical examination centers, and other departments at Shulan (Hangzhou) Hospital Affiliated to Zhejiang Shuren University Shulan International Medical College from October 2016 to May 2021 due to obesity, NAFLD, abnormal blood glucose and lipid metabolism. According to the presence or absence of NAFLD, the group was divided into obesity with NAFLD group and simple obesity group. 181 patients were finally eligible for this study, including 103 patients with obesity with NAFLD and 78 patients with obesity.

The inclusion criteria were as follows: 1) adults 18-95 years old, regardless of gender, Han Chinese; 2) BMI ≥ 28 kg/m^2^ defined as obese referring to the WHO standards for Asian populations (WHO Expert Consultation, 2004), NAFLD diagnosis meeting the diagnostic requirements of AASLD 2017 for NAFLD (Chalasani et al., 2018); 3) able to communicate, eat and move around normally.

The exclusion criteria were as follows: 1) age < 18 years old or > 95 years old; 2) viral liver disease, alcoholic liver disease, autoimmune disease, drug-related liver disease, metabolic liver disease, diabetes mellitus, acute myocardial infarction, severe renal failure, and malignancy; 3) critically ill patients with unstable vital signs; 4) the previous history of major gastrointestinal surgery; 5) diarrhea and other gastrointestinal disorders in the last 4 weeks; 6) having an infectious disease requiring antibiotic treatment; 7) history of antibiotics, micro-ecological regulators and other agents affecting gut microbiota in the last 4 weeks.

## Data collection

### Basic information

The patient’s height and weight are scaled by a professional, dressed lightly in the early morning. Systolic and diastolic blood pressure (SBP, DBP) was gauged in a quiet state and sitting position. Venous blood was drawn on a fasting stomach. Fasting blood glucose (FBG), glycated hemoglobin A1c (HbA1c), white blood cells, neutrophils (Neu), lymphocytes (Lym), monocytes (Mono), eosinophils sedimentation rate (ESR), total protein (TP), alanine aminotransferase (ALT), aspartate aminotransferase (AST), glutamyl transpeptidase (GGT), alkaline phosphatase (ALP), total bilirubin (TB), direct bilirubin (DB), indirect bilirubin (IB), adenosine deaminase (ADA), creatinine (Cr), uric acid (UA), urea nitrogen (BUN), total cholesterol (TC), triglycerides (TG), high-density lipoprotein (HDL), low-density lipoprotein (LDL), very low-density lipoprotein (VLDL), apolipoprotein A1 (apoA1), creatine kinase (CK), and lactate dehydrogenase (LDH) were measured by the hospital’s laboratory department. Ultrasonography was used to evaluate fatty liver.

### Gut dominant microbiota assay

Each patient’s mid-posterior feces were expelled into a sterile container, and 2.0 g of fresh feces samples were then collected from the medial middle using a sterile spoon into a disposable sterile feces stool collector. The samples were delivered to the lab right away, divided into 10 pieces of 0.2g each in fresh centrifuge tubes, and kept at -80° C within 30 minutes.

Each centrifuge tube should contain 1.3ml of Inhibit EX Buffer, a glass bead measuring 2 mm in diameter and 0.3g in mass, and a vortex oscillator set to 5000rpm for two 30s cycles. After that, the supernatant was centrifuged for 3 minutes at 15000 rpm twice. Follow the manufacturer’s instructions when using the QIAamp Fast DNA Stool Mini Kit to extract DNA (Ma et al., 2011). A PCR quantitative assay was used to examine the quantity of DNA (ABI ViiA7, USA). Utilizing c-Atopo-F (5’-GGGTTGAGAGACCGACC-3’), c-Atopo-R (5’-CGGRGCTTCTTCTGCAGG-3’), Sg-Clept-F (5’-GCACAAGCAGTGGAGT-3’), Sg-Clept-R3 (5’-CTTCCTCCGTTTTGTCAA-3’) (Matsuki et al., 2004), Lac-F (5’-AGCAGTAGGGAATCTTCCA-3’), Lac-R (5’-ATTYCACCGCTACACATG-3’), Eco-F (5’-CATTGACGTTACCCGCAGAAGAAGC-3’), Eco-R (5’-CTCTACGAGACTCAAGCTTGC-3’) (He et al., 2013), Bac-F (5’-GAAGGTCCCCCACATTG-3’), Bac-R (5’-CAATCGGAGTTCTTCGTG-3’), Enco-F (5’-AACCTACCCATCAGAGGG-3’), Enco-R (5’-GACGTTCAGTTACTAACG-3’), Pfra-F (5’-GATGGCCTCGCGTCCGATTAG-3’), Pfra-R (5’-CCGAAGACCTTCTTCCTCC-3’), Bif-F (5’-GGGTGGTATGCCGGATGTAA-3’), Bif-R (5’-GCCATGGACTTTCACACC-3’) (Bartosch et al., 2004), Clos-F (5’-CGGTACCTGACTAAGAAGC-3’), Clos-R (5’-AGTTT(C/T)ATTCTTGCGAACG-3’) (Rinttilä et al., 2004), CI-F (5’-TACCHRAGGAGGAAGCCAC-3’), and CI-R (5’-GTTCTTCCTAATCTCTACGCAT-3’) (Song et al., 2004), samples were amplified by PCR (Atopobium cluster, Clostridium leptum, Lactobacillus, Enterobacteriaceae, Bacteroides, Enterococcus, F. prausnitzii, Bifidobacterium, Eubacterium rectale, Clostridium butyricum). The PCR reaction system was 20μl, which includes STBR Green PCR Master Mix 10μl, Forward primer 0.4μl, Reverse primer 0.4μl, DNA Template 2μl, and Rnase Free H2O 7.2μl. The primers were subjected to 40 cycles of pre-denature at 95° C for 3 min, denature at 95° C for the 30s, annealing at 58° C for 40s, and extension at 72° C for 5 min. To prevent interference from the secondary structure and primer-dimer on the detection, fluorescence intensity was measured for 10 seconds at the end of each cycle. By integrating the Ct values of the samples acquired from the test, the initial copy number of the target gene in each sample was determined against the standard curve in three replicate wells for each sample.

## Statistical methodology

The general clinical information of the patients was extracted and entered by Python 3.8.6 software using regular expressions. SPSS 26.0 statistical software was applied for data analysis, and GraphPad Prism 9, R 4.2.2, and Origin 2022 software were applied for graphing. Measures that conformed to normal distribution were expressed by Mean ± SD, and a t-test was used for comparison between the two; measures that did not conform to normal distribution were expressed by M (Q25, Q75), and a non-parametric test was used for comparison between two groups. Assessment of differences and similarities in the gut dominant microbiota between obesity with NAFLD and simple obesity was achieved using PCA. Single-factor logistic regression analysis was performed to screen the influencing factors of obesity with NAFLD, and multi-factor logistic regression was performed to further analyze the risk factors and protective factors of obesity with NAFLD. To search the variables associated with gut dominant microbiota, univariate as well as multivariate correlation regression was applied to explore the factors affecting gut dominant microbiota.

## Results

### Basic clinical information

This study included 181 patients with BMI ≥ 28 kg/m^2^, divided into 103 cases in the obesity with NAFLD group and 78 cases in the simple obese group according to the presence or absence of nonalcoholic fatty liver. A total of 117 men and 64 women, 103 were in the obesity with NAFLD group (56.9%) and 78 were in the simple obesity group. The mean age was 52.90 ± 1.26 years, and 64.6% were male. The prevalence of concomitant NAFLD in obese patients was 56.9%. **Table 1** contains demographic, inflammatory, and metabolic indicators for the two groups. As shown in **Table 1**, there were no significant differences in age and sex between the two groups, so the two populations were comparable. Compared to the simple obese group, the obesity with NAFLD group had higher levels of BMI, systolic blood pressure, lymphocytes, ALT, AST, GGT, and CK (*p*-value < 0.05). In contrast, other inflammatory indicators including leukocytes, neutrophils, monocytes, eosinophils, basophils, CRP, and ESR were not statistically significant. In addition, there was no significant difference between the obesity with NAFLD group and the simple obesity group for bilirubin, uric acid, creatinine, and lipid-related indices.

**Table 1:** Comparison of general data of obesity with NAFLD and simple obesity.

	Total n=181	Simple obesity n=78	Obesity with NAFLD n=103	Z or t value	*P*
Age	52.90±1.26	55.42±1.92	50.98±1.67	0.13	0.718
Male	117(64.6%)	49(62.8%)	68(66.0%)	0.20	0.656
BMI	29.41(28.58,31.16)	29.09(28.40,30.93)	29.62(28.75,31.64)	2.15	0.032
SBP	135.82±1.341	135.00±2.25	136.45±1.63	0.54	0.040
DBP	82.44±1.24	81.61±1.56	83.12±1.27	1.28	0.259
Leukocyte	6.05(5.20,7.53)	6.00(5.15,7.33)	6.55(5.48,8.40)	1.88	0.060
Neu	3.55(2.60,4.53)	3.65(2.88,4.53)	3.65(2.98,4.90)	0.70	0.486
Lym	1.90(1.50,2.40)	1.80(1.40,2.20)	2.10(1.60,2.63)	3.10	0.002
Mono	0.500(0.40,0.60)	0.50(0.40,0.50)	0.45(0.40,0.60)	0.75	0.455
Eos	0.12(0.07,0.19)	0.11(0.08,0.18)	0.13(0.07,0.19)	1.38	0.168
Baso	0.30(0.20,0.40)	0.300(0.20,0.40)	0.300(0.20,0.40)	0.99	0.323
CRP	2.19±0.11	1.55(0.90,2.88)	2.35±0.14	1.82	0.068
ESR	8.00(4.00,14.75)	9.00(3.00,14.00)	7.00(5.00,15.00)	0.15	0.879
HbA1c	5.60(5.40,6.40)	5.50(5.30,6.20)	5.70(5.40,6.50)	1.85	0.065
FBG	5.50(4.93,6.00)	5.14(4.78,6.03)	5.56(5.06,6.00)	1.67	0.095
TP	68.69±0.56	68.47±0.89	69.20(64.33,71.98)	0.54	0.591
ALT	29.00(18.00,51.00)	23.00(14.75,43.50)	34.00(24.00,63.00)	3.29	0.001
AST	23.00(19.00,32.00)	21.00(16.00,30.00)	24.00(20.00,32.00)	2.15	0.032
GGT	36.00(23.00,66.00)	27.00(16.00,72.50)	40.00(25.00,66.00)	2.24	0.025
ALP	63.00(53.00,77.00)	64.00(51.75,76.00)	63.00(53.00,79.00)	0.27	0.788
TB	10.50(8.00,14.00)	11.00(9.00,14.25)	10.00(7.00,14.00)	1.23	0.220
DB	3.00(2.00,5.00)	3.00(2.00,4.00)	3.00(2.00,5.00)	1.04	0.299
IB	7.00(6.00,10.00)	8.00(6.00,10.00)	7.00(5.00,10.00)	1.20	0.230
ADA	10.00(8.00,12.00)	10.00(8.00,12.00)	10.00(8.00,13.00)	0.47	0.637
Cr	72.51±3.40	69.00(57.00,80.00)	65.00(57.00,76.00)	1.41	0.159
UA	379.08±8.24	355.56±11.42	374.50(334.50,429.75)	2.15	0.031
BUN	5.19(4.22,6.30)	5.52(4.46,6.53)	5.09(4.15,5.89)	1.70	0.088
TC	4.78±0.12	4.46±0.22	4.99±0.12	3.46	0.065
TG	2.41±0.14	1.68(1.18,2.64)	1.93(1.36,2.74)	1.44	0.150
HDL	1.09±0.02	1.13±0.03	1.05±0.03	1.93	0.167
LDL	2.88±0.07	2.76±0.10	2.94±0.09	1.00	0.318
VLDL	0.77(0.54,1.17)	0.77(0.48,1.21)	0.83(0.58,1.14)	0.95	0.343
apoA1	1.10(0.98,1.28)	1.14(0.99,1.32)	1.06(0.96,1.25)	1.63	0.102
CK	87.00(69.25,125.00)	83.00(54.50,118.50)	96.60(72.00,146.00)	2.13	0.034
LDH	192.50(170.00,221.50)	188.00(159.50,208.00)	193.00(172.00,227.00)	1.37	0.171

### Differences in the composition of the gut dominant microbiota

**Figure 1:**
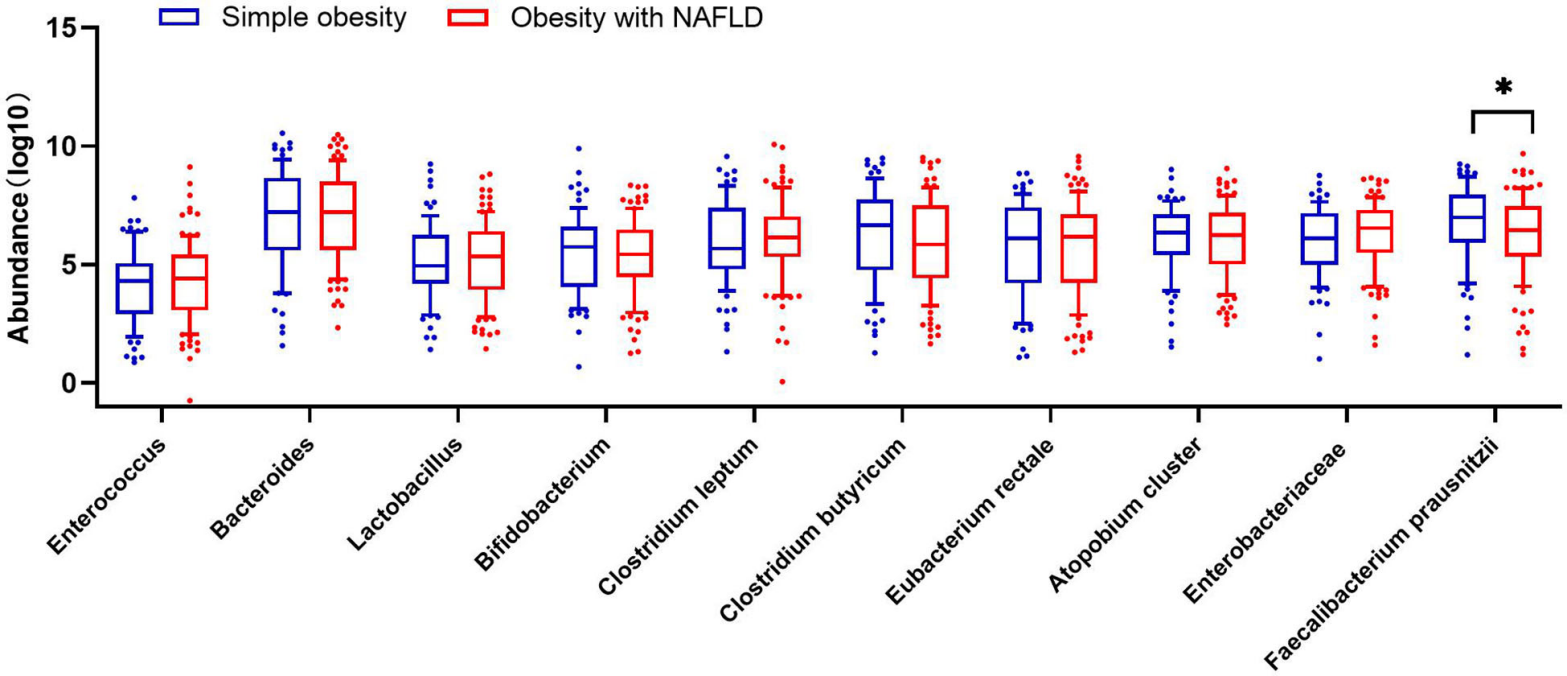
The number of dominant gut microbiota colonies in 181 patients with obesity with NAFLD and simple obesity. The symbol * represents p-value less than 0.05.

**Figure 2:**
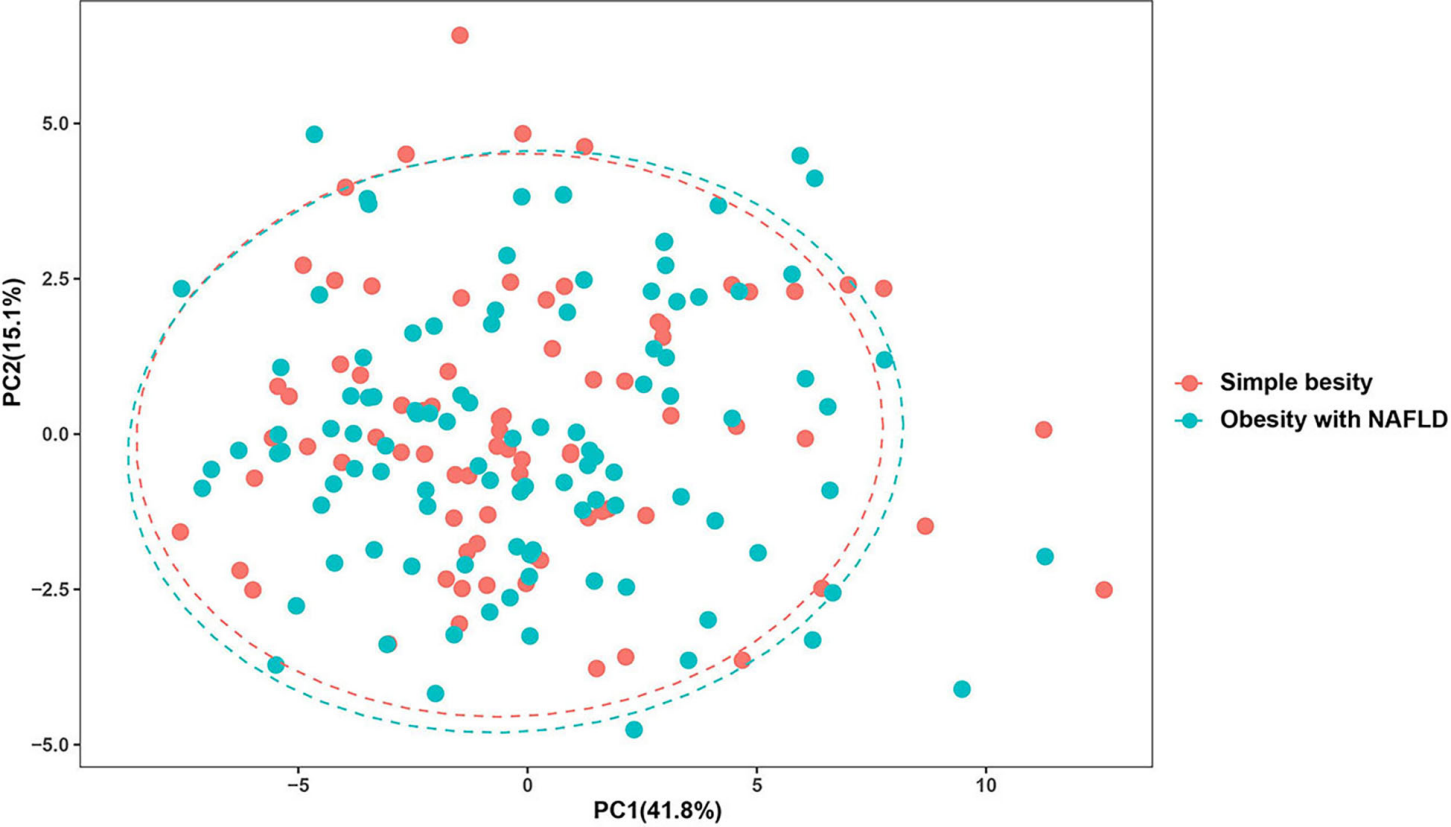
Principal component analysis of the dominant gut microbiota in obesity with NAFLD and simple obesity.

**Figure 3:**
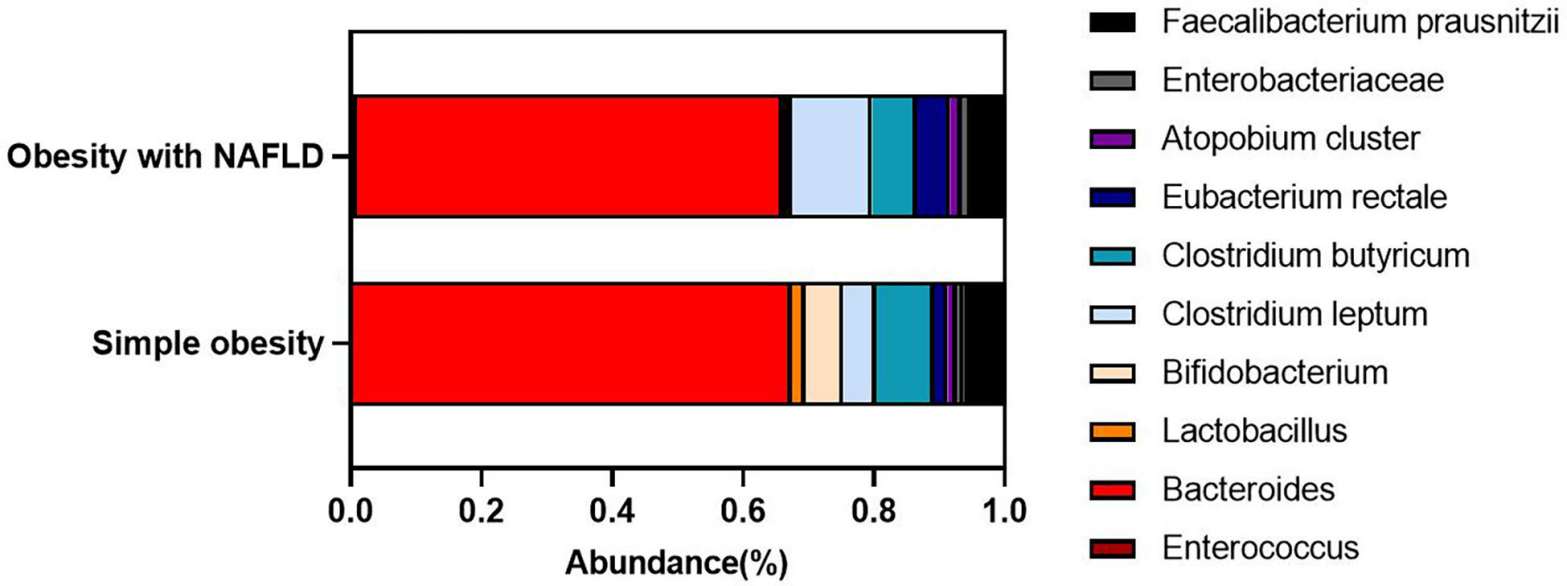
Stacked Bar chart of the dominant gut microbiota in obesity with NAFLD and simple obesity.

**Figure 4:**
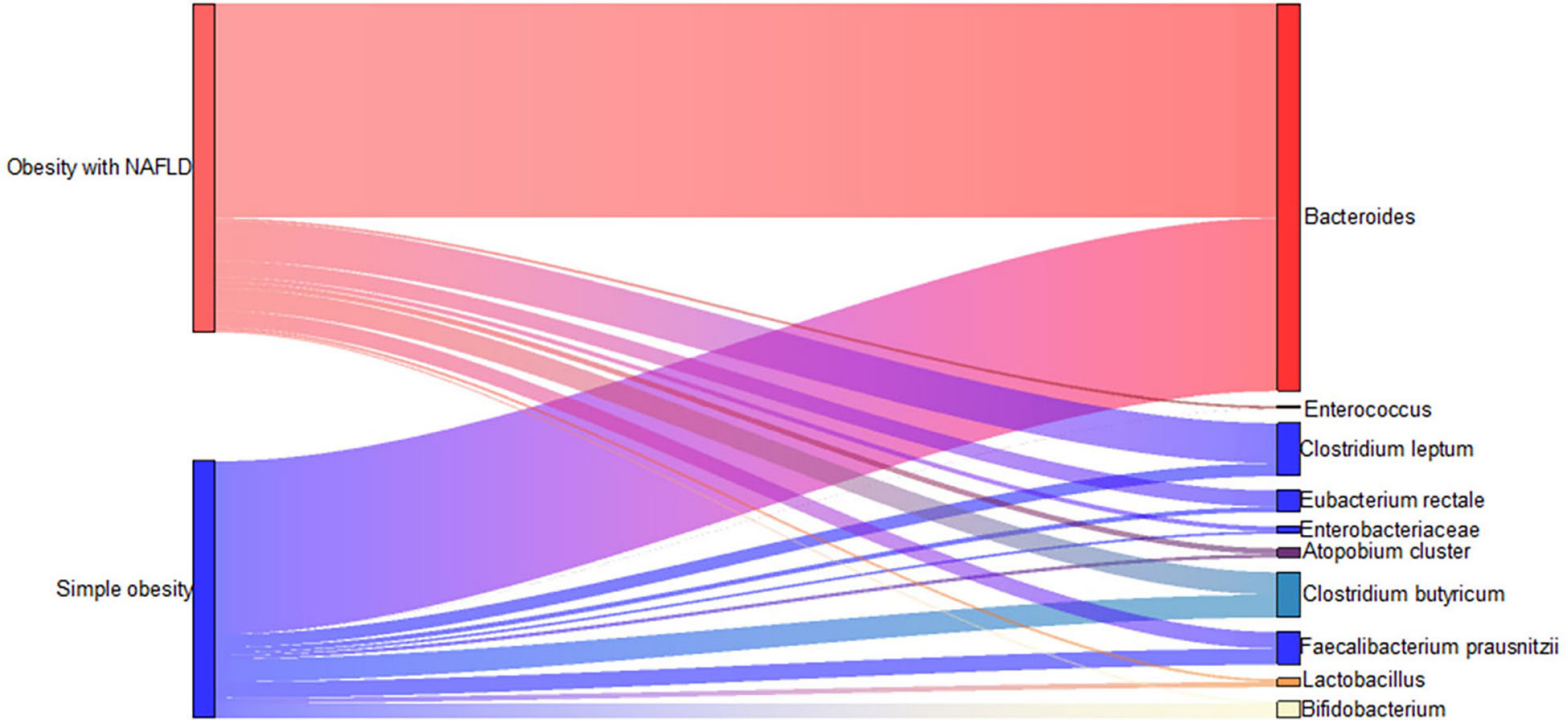
Sankey plots of the dominant gut microbiota in obesity with NAFLD and simple obesity.

DNA extracted from feces was used to quantify the number of colonies of 10 dominant gut microbiota by fluorescence. As shown in **Table 2** and **Figure 1**, the number of colonies of *F. prausnitzii* colonies was significantly lower in the obesity with NAFLD group than in the simple obesity group (*p*-value < 0.05). By principal component analysis (**Figure 2**), PC1 and PC2 contributed 56.9%. There was a large overlap between the graphs of the two groups, as a result, there was a similarity in the gut dominant microbiota between the obesity with NAFLD and simple obesity groups. We applied a Stacked Bar chart (**Figure 3**) and Sankey plots (**Figure 4**) to visualize and compare the composition of the gut dominant microbiota in the obesity with NAFLD and simple obesity groups, and both groups had the highest percentage of *Bacteroides*, over 65%. The obesity with NAFLD group *Bacteroides*, *Clostridium leptum*, and *Clostridium butyricum* accounted for more than 80%, while the simple obesity group *Bacteroides*, *Clostridium butyricum*, and *F. prausnitzii* accounted for more than 80%.

**Table 2:** Comparison of the gut dominant microbiota in two groups of obesity with NAFLD and simple obesity.

	Total n=181	Simple obesity n=78	Obesity with NAFLD n=103	*P*
*Enterococcus*	4.22±0.12	4.15±0.18	4.27±0.17	0.734
*Bacteroides*	7.12±0.15	7.15±0.25	7.10±0.18	0.239
*Lactobacillus*	5.20±0.12	5.20±0.19	5.19±0.16	0.482
*Bifidobacterium*	5.37±0.13	5.42±0.21	5.33±0.16	0.139
*Clostridium leptum*	6.05±0.13	6.02±0.20	6.08±0.17	0.460
*Clostridium butyricum*	6.06±0.15	6.63(4.68,7.83)	5.92±0.19	0.176
*Eubacterium rectale*	5.72±0.15	6.37(4.12,7.44)	5.71±0.20	0.849
*Atopobium cluster*	6.31(5.16,7.14)	6.39(5.41,7.12)	6.24(5.00,7.19)	0.766
Enterobacteriaceae	6.47(5.28,7.22)	6.15(5.12,7.20)	6.54(5.49,7.31)	0.141
*Faecalibacterium prausnitzii*	6.47±0.13	7.03(5.94,8.05)	6.27±0.17	0.0499

**Figure 5:**
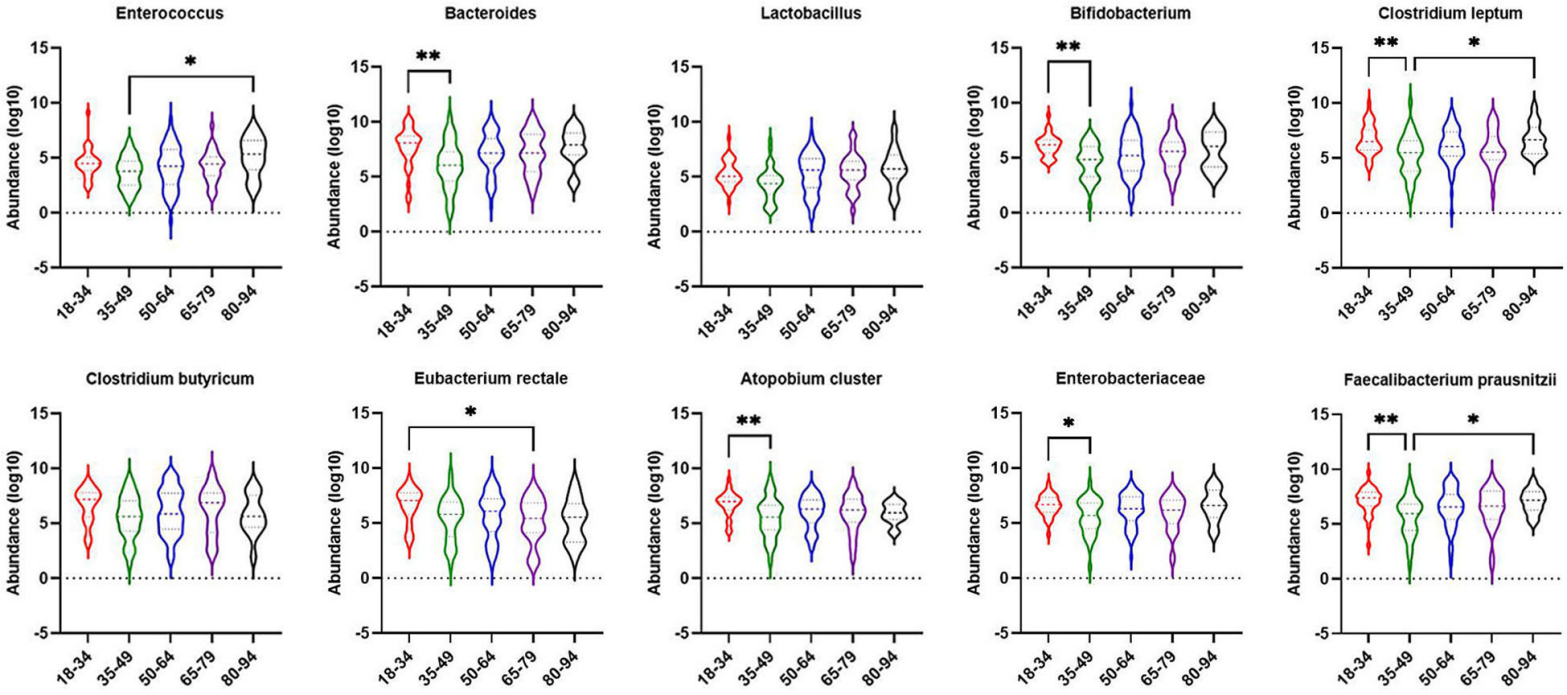
Comparison of dominant gut microbiota in different age groups of obesity with NAFLD and simple obesity populations. The symbol * represents p-value less than 0.05. And the symbol ** represents p-value less than 0.01.

The obese combined NAFLD and the simple obese population were stratified by age to compare differences in gut dominant microbiota across age strata. As shown in **Figure 5**, *Bacteroides* (*p*-value < 0.01), *Bifidobacterium* (*p*-value < 0.01), *Clostridium leptum* (*p*-value < 0.01), *Atopobium cluster* (*p*-value < 0.01), Enterobacteriaceae (*p*-value < 0.05), *F. prausnitzii* (*p*-value < 0.01) were significantly higher for those aged 18–34 years than for those aged 35–49 years. *Clostridium butyricum* (*p*-value < 0.05) was significantly higher for those aged 18–34 years than for those aged 65–79 years. *Enterococcus*, *Clostridium leptum*, and *F. prausnitzii* were significantly lower for those aged 35–49 years than for those aged 80–94 years (*p*-value < 0.05).

### Single-factor logistic regression analysis of obesity with NAFLD

To investigate the factors influencing obese patients comorbid for NAFLD, we performed single-factor logistic regression analysis for indicators of BMI, age, inflammatory biomarkers, and metabolic indicators respectively. **Table 3** shows the indicators with *p*-values less than 0.1.

**Table 3:** Single-factor logistic regression analysis of obesity with NAFLD (*P* < 0.1).

	OR(95% CI)	*P*
BMI	1.105(0.989-1.234)	0.079
Age	0.985(0.967-1.002)	0.084
Lym	2.202(1.336-3.628)	0.002
CRP	1.348(0.950-1.912)	0.094
UA	1.004(1.001-1.007)	0.021
TC	1.253(1.021-1.536)	0.031
HDL	0.369(0.128-1.067)	0.066
ApoA1	0.360(0.107-1.205)	0.097
CK	1.007(1.001-1.014)	0.033
*Faecalibacterium prausnitzii*	0.854(0.712-1.025)	0.091

### Multi-factor logistic regression analysis of obesity with NAFLD

**Figure 6:**
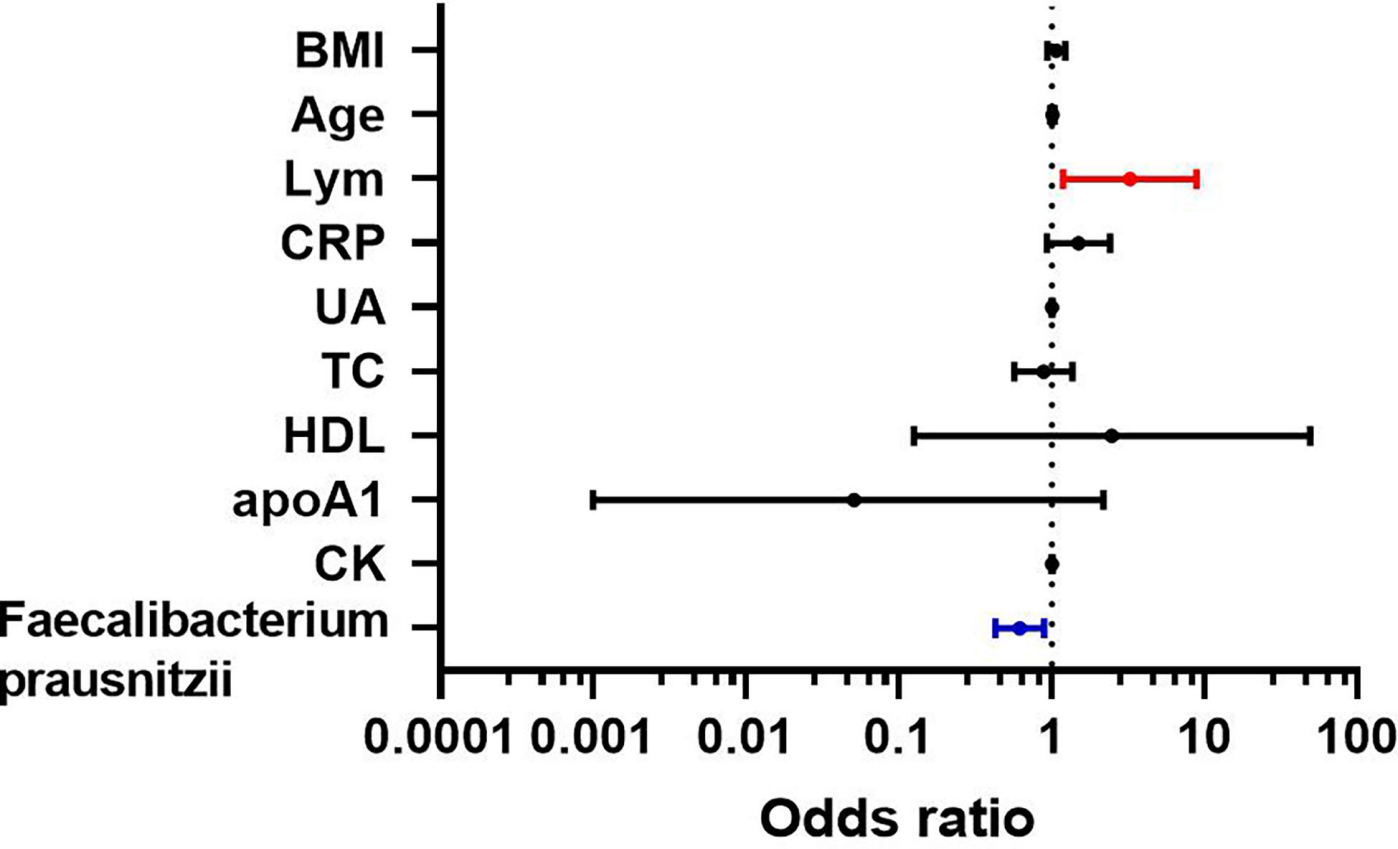
Multi-factor logistic regression analysis of obesity with NAFLD.

The indicators of BMI, age, lymphocytes, CRP, UA, TC, HDL, ApoA1, CK, and *F. prausnitzii*, which were analyzed by single-factor logistic regression with *p*-value < 0.1, were included in the multi-factor logistic regression equation. The Hosmer-Lemeshow test score of 0.709 and the model coefficient Omnibus test of significance of 0.011 both indicate that the model is generally relevant. *F. prausnitzii* (OR = 0.618, 95%CI 0.427-0.893, *p*-value = 0.010) may be a protective factor against obesity with NAFLD, but lymphocytes (OR = 3.237, 95% CI 1.184-8.854, *p*-value = 0.022) may be a risk factor for obesity with NAFLD (**Figure 6**). In addition, ALT, AST, and GGT were not risk factors for the development of NAFLD in obese patients (*p*-value > 0.05).

**Figure 7:**
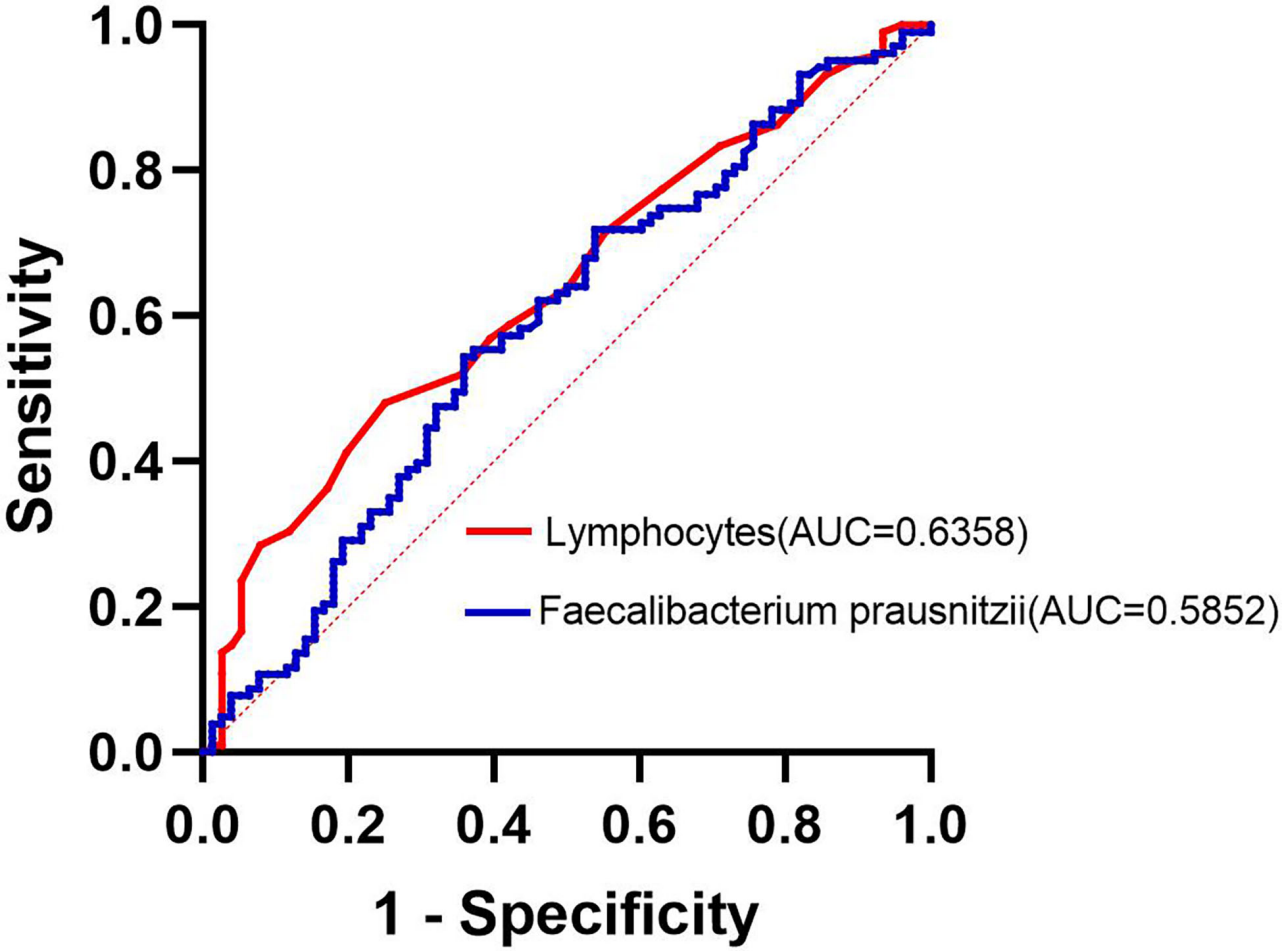
Lymphocytes and *F. prausnitzii* ROC curve analysis for predicting the development of NAFLD in obese patients.

To Compare the ability of lymphocytes and *F. prausnitzii* to predict the development of NAFLD in obese patients, ROC curves were drawn in **Figure 7**. AUC for lymphocytes was 0.636 with 48.0% sensitivity and 75.0% specificity at a cutoff of 2.15 10^9^/L. AUC for *F. prausnitzii* was 0.585 with 55.4% sensitivity and 64.1% specificity at a cutoff of 6.60 log_10_CFU/g (**Figure 7**).

### Univariate correlation analysis between *F. prausnitzii* and each indicator

**Figure 8:**
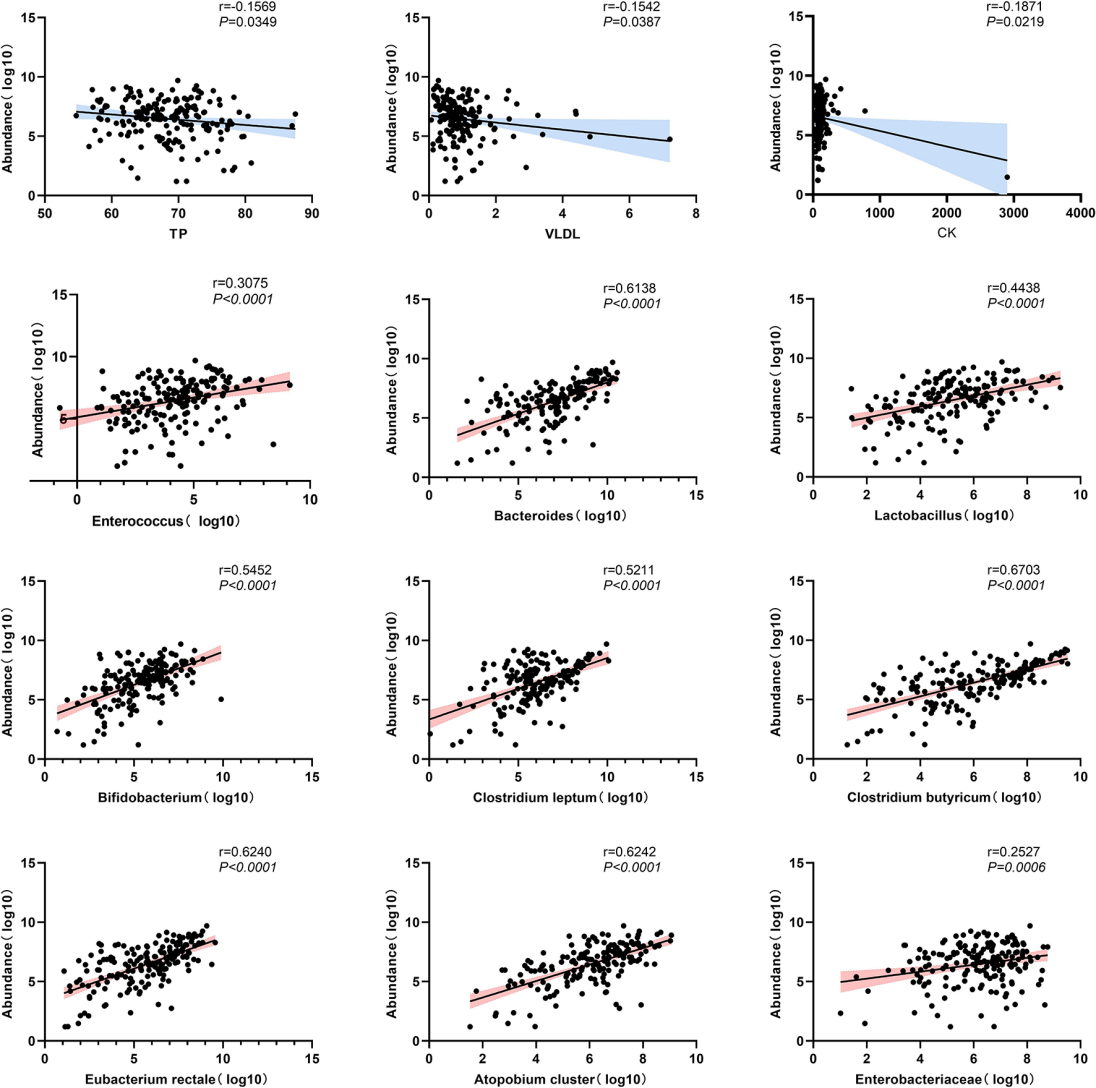
Scatterplot of univariate correlation analysis between *F. prausnitzii* and factors (*p*-value < 0.05).

**Figure 9:**
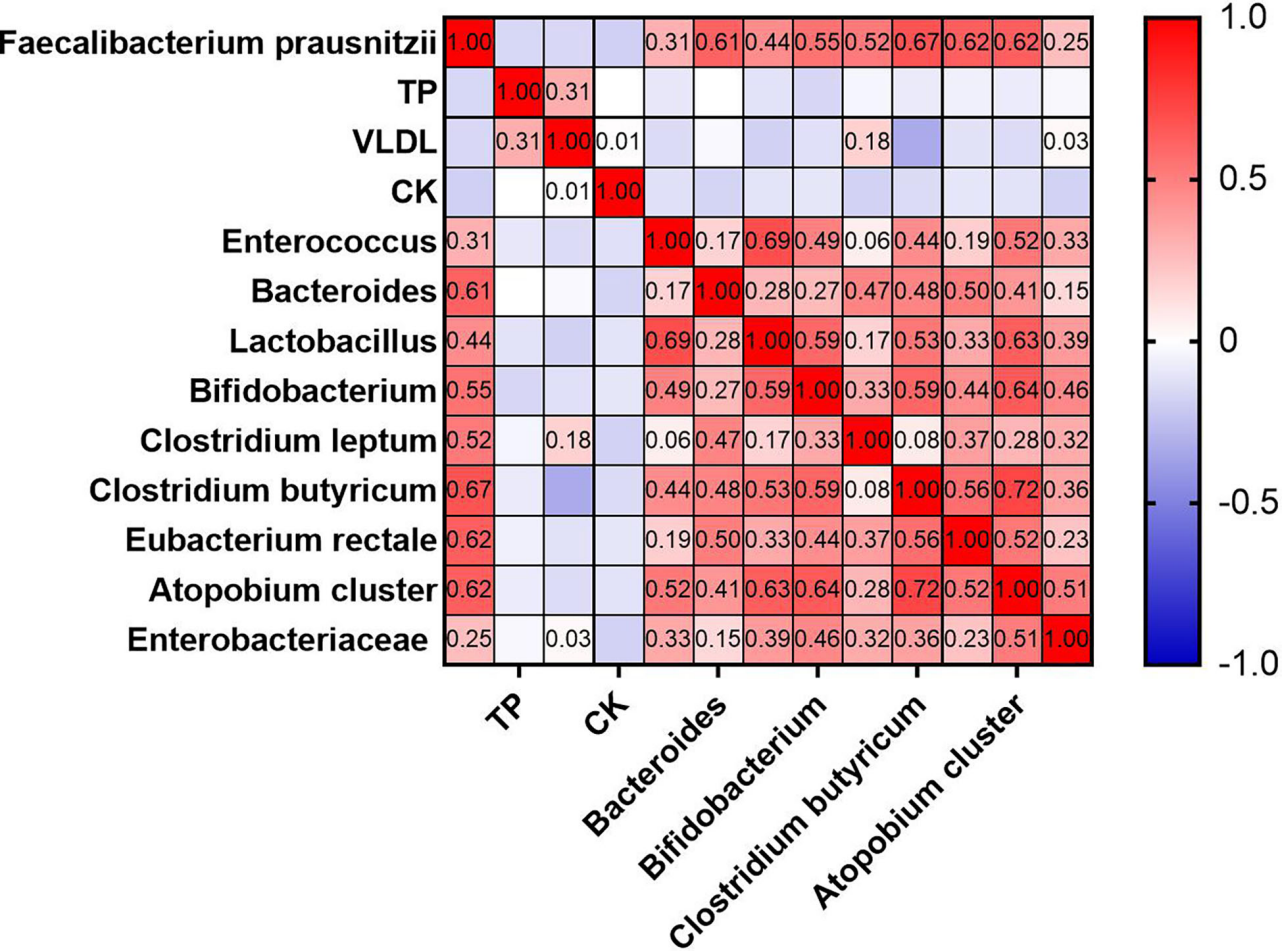
Heat map of univariate correlation analysis between *F. prausnitzii* and factors (*p*-value < 0.05).

Inflammatory biomarkers and metabolic indicators and gut dominant microbiota were analyzed separately with univariate correlation analysis. The *p*-values less than 0.05 were as follows (**Figure 8**). TB, VLDL, and CK were negatively correlated with *F. prausnitzii* (*p*-value < 0.05). *Enterococcus* (*p*-value < 0.001), *Bacteroides* (*p*-value < 0.001), *Lactobacillus* (*p*-value < 0.001), *Bifidobacterium* (*p*-value < 0.001), *Clostridium leptum* (*p*-value < 0.001), *Clostridium butyricum* (*p*-value < 0.001), *Eubacterium rectale* (*p*-value < 0.001), *Atopobium cluster* (*p*-value < 0.001) and Enterobacteriaceae (*p*-value < 0.01) were positively correlated with *F. prausnitzii*. In contrast, inflammatory indicators such as leukocytes, neutrophils, monocytes, eosinophils, basophils, CRP, and ESR were not correlated with *F. prausnitzii*. We plotted a heat map to more visually show the correlation between *F. prausnitzii* and factors, as shown in **Figure 9**.

### Multivariate linear regression analysis of *F. prausnitzii* and factors

**Figure 10:**
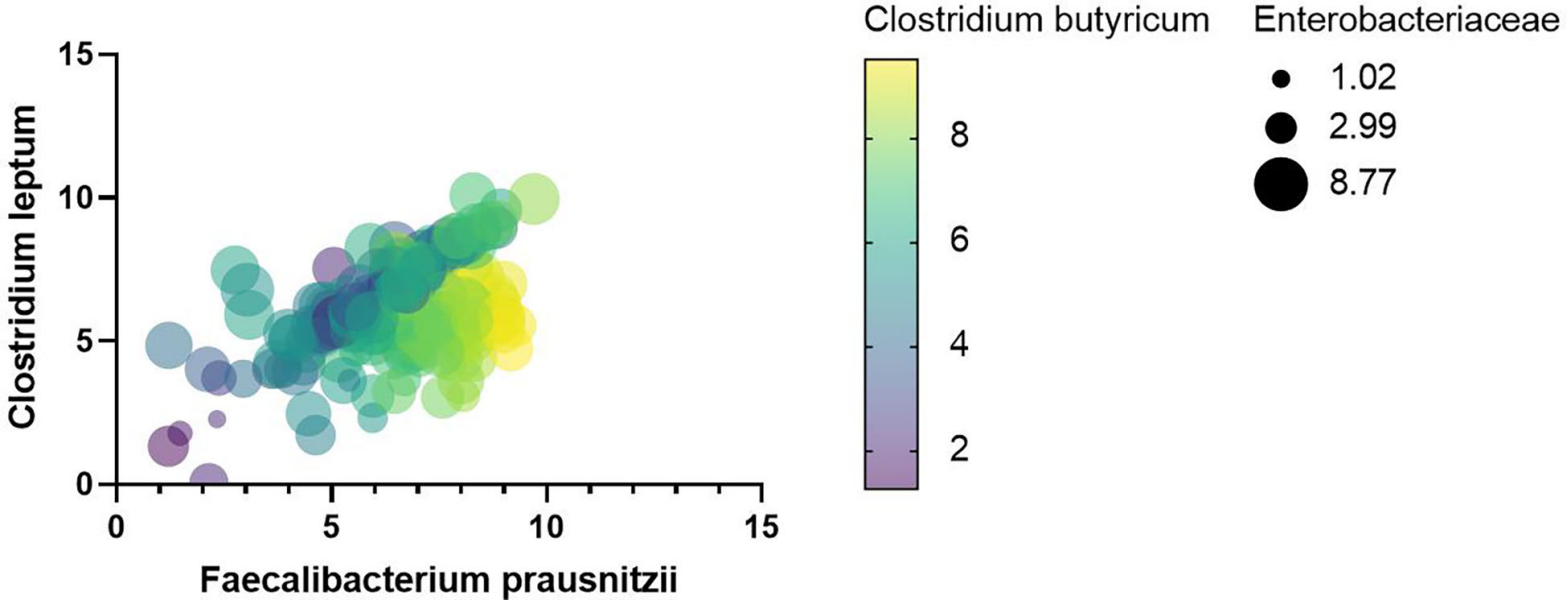
Heat map between *F. prausnitzii* and indicators with *p*-values less than 0.001 for multivariate regression analysis.

After univariate correlation analysis, indicators with *p*-values less than 0.05 were included in the multivariate linear regression analysis, containing TB, VLDL, CK, *Enterococcus*, *Bacteroides*, *Lactobacillus*, *Bifidobacterium*, *Clostridium leptum*, *Clostridium butyricum*, *Eubacterium rectale*, *Atopobium cluster*, and Enterobacteriaceae. The R^2^ of the multivariate linear regression was 0.738 suggesting a good fit, the Durbin-Watson value of 1.2 suggested good sample independence, the VIF was less than 5 suggesting no multicollinearity, and normally distributed residuals. The effect of *Bacteroides* (*p*-value < 0.05), *Clostridium leptum* (*p*-value < 0.001), *Clostridium butyricum* (*p*-value < 0.001), and *Eubacterium rectale* (*p*-value < 0.05) on *F. prausnitzii* was positive, yet Enterobacteriaceae was negative (*p*-value < 0.001) (**Table 4**). As shown in **Figure 10**, the more yellow the color of the *Clostridium butyricum* image, the smaller the graph of Enterobacteriaceae, the higher the level of *Clostridium leptum* colonies, and then the higher the level of *F. prausnitzii* colonies.

**Table 4:** Multivariate linear regression analysis of *Faecalibacterium prausnitzii* and metabolic indicators and other gut microbiota.

	r	*P*
TP	-1.140	0.256
VLDL	0.585	0.560
CK	-0.745	0.457
*Enterococcus*	0.189	0.851
*Bacteroides*	2.294	0.023
*Lactobacillus*	0.323	0.747
*Bifidobacterium*	0.915	0.362
*Clostridium leptum*	6.132	<0.001
*Clostridium butyricum*	5.420	<0.001
*Eubacterium rectale*	2.117	0.036
*Atopobium cluster*	1.740	0.084
Enterobacteriaceae	-3.448	<0.001

## Discussion

It is well-accepted that NAFLD and obesity are closely associated, but thin persons can also develop NAFLD (Younes et al., 2022). The pathogenesis of obesity includes inactivity, dyslipidemia, insulin resistance, inflammation, and gut microbiota (Meijnikman et al., 2018; Truax et al., 2018; Depommier et al., 2019; Kazak and Cohen, 2020; Wu et al., 2021). Obesity-related mechanisms frequently contribute to the development of NAFLD. For instance, dyslipidemia, insulin resistance, and gut microbiota have been considered causative mechanisms of NAFLD (Friedman et al., 2018; Bauer et al., 2022). Both obesity with NAFLD and obesity are associated with gut microbiota. *Desulfovibro* is growing whereas Clostridia is declining in the obese population, and Bacteroidetes may increase or decrease (Liu et al., 2017; Wang and Hooper, 2019; Vieira-Silva et al., 2020). *Escherichia*, *Dorea*, and *Peptoniphilus* significantly increased in NAFLD, but *Bacteroides* (Cheng et al., 2022), *F. prausnitzii *(Pettinelli et al., 2022), *Eubacterium* and *Prevotella* (Del Chierico et al., 2017; Hoyles et al., 2018) significantly decreased. Similarly, *F. prausnitzii* was significantly lower in obesity with NAFLD than in simple obesity. The distinction was that, as compared to simple obesity, obesity with NAFLD did not result in a discernible decline in *Bacteroides*. There hasn’t been any thorough research on the gut microbiota traits of obesity with NAFLD. In this study, we explored that the gut dominant microbiota was similar between obesity with NAFLD and simple obesity, but the *F. prausnitzii* colony number was much lower in the obesity with NAFLD than in the simple obesity (*p*-value < 0.05). The proportion of *Bacteroides* is highest in both groups of obesity with NAFLD and simple obesity, at over 65%. *Bacteroides*, *Clostridium leptum*, and *Clostridium butyricum* accounted for more than 80% of the cases of obesity with NAFLD, whereas *Bacteroides*, *Clostridium butyricum*, and *F. prausnitzii* accounted for more than 80% of the cases of simple obesity.

Uncertainty exists regarding how gut microbiota affects obesity and NAFLD. The binding of lipids encoded by gut flora to G-protein-coupled receptors that control metabolic hormones and glucose homeostasis is one possible mechanism by which gut microbiota may contribute to NAFLD (Cohen et al., 2017). Another possibility is that gut gram-negative bacterial cell wall LPS may reach the liver through an improperly functioning gut barrier, triggering an inflammatory reaction in the liver (Brandl and Schnabl, 2017). In addition, bile acids and gut microbial metabolites may play possible roles in NAFLD (Jiao et al., 2018; Canfora et al., 2019; Chu et al., 2019; Aron-Wisnewsky et al., 2020a; Ortiz-López et al., 2022). Lymphocytes may be a risk factor for obesity with NAFLD, yet *F. prausnitzii* may act as a barrier against NAFLD in obese patients in our research.

A key factor in the pathogenesis of NAFLD is chronic low-grade inflammation, which is linked to altered gut microbiota and elevated lymphocyte activation (Ortiz-López et al., 2022). Lymphocyte accumulation is seen in the liver of NAFLD patients, and lymphocytes can trigger oxidative stress leading to NAFLD (Bruzzì et al., 2018; Sutti and Albano, 2020; Ortiz-López et al., 2022). Through the release of interleukin 17, lymphocytes may alter the gut microbiota, which both causes and worsens NAFLD (Gomes et al., 2016; Douzandeh-Mobarrez and Kariminik, 2019). For instance, *segmented filamentous bacteria* that increase interleukin 17 production, colonized mice and aggravated the hepatocellular damage brought on by obesity (Harley et al., 2014). Furtherly, hepatocyte death and fibrosis may be connected in a significant way *via* activation of the hepatocyte inflammasome (Friedman et al., 2018). This shows the theoretical justification for our study’s presentation of obesity with NAFLD as having greater lymphocytes than simple obesity. According to our investigation, lymphocytes may also be a risk factor for obesity with NAFLD, yet *F. prausnitzii* was an independent protective factor. Therefore, it may be advantageous to raise *F. prausnitzii* levels while lowering lymphocyte levels in obese patients to prevent the onset of NAFLD. Compared to models that just employ host genetic and environmental information, microbiota data dramatically increases the prediction accuracy for several human variables (Rothschild et al., 2018). Our analysis revealed that lymphocytes and *F. prausnitzii* contribute to predicting the development of NAFLD in obese patients.

Reduced abundance of *F. prausnitzii* has been related to a variety of diseases, including obesity (Haghikia et al., 2022), type 2 diabetes (Gurung et al., 2020), inflammatory bowel disease (Sartor and Wu, 2017; Kelly and Ananthakrishnan, 2019), colorectal cancer (Kang and Martin, 2017). Meanwhile, Vallianou et al. found a significant reduction in *F. prausnitzii* (Vallianou et al., 2021). Thus, increasing *F. prausnitzii* levels may also be beneficial in preventing the development of NAFLD in obese patients. Recently, a study demonstrated that mice on a high-fat diet given *F. prausnitzii* suspensions made in an anaerobic environment have decreased liver fat (Munukka et al., 2017). *F. prausnitzii* is anaerobic and dies quickly after being exposed to air, so it is challenging to replenish *F. prausnitzii* directly. Interactions between gut microbiota are possible, the theoretical underpinnings of gut microbiota interactions include metabolite stimulation, direct stimulation or inhibition, and host effect (Sanders et al., 2019; Suzuki and Ley, 2020). Previously, it was found that *B. uniformis* and *B. thetaiotaomicron* may work in conjunction to reduce weight (Liu et al., 2017). It has previously been demonstrated that different gut microbiota encourages the growth of *F. prausnitzii*, for instance, B. thetaiomicron and *F. prausnitzii* co-cultured organisms create butyric acid, which aids in the growth of *F. prausnitzii* (Lopez-Siles et al., 2017). Therefore, it might be also possible to raise the level of *F. prausnitzii* by promoting the number of *Bacteroides*, *Clostridium leptum*, *Clostridium butyricum*, and *Eubacterium rectale* colonies while decreasing the number of Enterobacteriaceae colonies.

This study was a retrospective case-control study. Thus, causality could not be verified. The research population was limited to one hospital and there was no multicenter association, which may not give a full picture of the overall situation. Stool samples are frozen-stored, which over time may skew microbiota data. The gut microbiota analysis used fluorescence quantification of the colony counts of the ten dominant gut microbiotas. For a comprehensive perspective of the microbiota, qPCR has limitations, therefore it could not analyze the effect of other gut microbiota on obesity with NAFLD. There are newer, more accurate methods for carrying out these kinds of investigations based on DNA sequencing and bioinformatics, such as 16S based metagenomic amplicon sequencing and/or shotgun metagenomics. Subsequent animal studies are proposed to be refined to explore whether increased lymphocytes, as well as decreased *F. prausnitzii*, may increase the risk of developing NAFLD in obese patients.

## Conclusion

The gut dominant microbiota was similar between obesity with NAFLD and simple obesity. However, the quantity of *F. prausnitzii* colonies was much lower in obesity with NAFLD compared to simple obesity. *F. prausnitzii* was an independent protective factor for obesity with NAFLD, while lymphocytes were an independent risk factor. In addition, lymphocytes and *F. prausnitzii* contribute to predicting the development of NAFLD in obese patients.

## Data availability statement

The original contributions presented in the study are included in the article/supplementary materials. Further inquiries can be directed to the corresponding author/s.

## Ethics statement

This study was reviewed and approved by the ethics committee of Shulan (Hangzhou) Hospital Affiliated to Zhejiang Shuren University Shulan International Medical College. The patients/participants provided their written informed consent to participate in this study.

## Author contributions

M-ZX conceptualized and designed the study. L-TJ collected samples, analyzed the data, and generated the figures. M-ZX and L-TJ both contributed to the first draft of the manuscript. M-ZX revised the manuscript. All authors contributed to the article and approved the submitted version.
